# A Spatiotemporal Tunable Filter Array Chip for Video-Rate Hyperspectral Imaging

**DOI:** 10.1021/acs.nanolett.4c05603

**Published:** 2025-02-03

**Authors:** Zijian Lin, Tingbiao Guo, Zhi Zhang, Yuan Zhang, Haochen Chu, Yijia Zeng, Xiao Chen, Nan Wang, Ruili Zhang, Sailing He

**Affiliations:** † Centre for Optical and Electromagnetic Research, College of Optical Science and Engineering, Zhejiang University, Hangzhou, 310058, People’s Republic of China; ‡ Zhejiang Engineering Research Center for Intelligent Medical Imaging, Sensing and Non-invasive Rapid Testing, Taizhou Hospital, Zhejiang University, Taizhou 318000, People’s Republic of China; § Shanghai Institute for Advanced Study, Zhejiang University, Shanghai 201203, People’s Republic of China; ∥ National Engineering Research Center for Optical Instruments, Zhejiang University, Hangzhou 310058, People’s Republic of China; ⊥ Department of Electromagnetic Engineering, School of Electrical Engineering, KTH Royal Institute of Technology, Stockholm, SE-100 44, Sweden

**Keywords:** hyperpectral imaging, chip, tunable filter, spatiotemporal, video rate

## Abstract

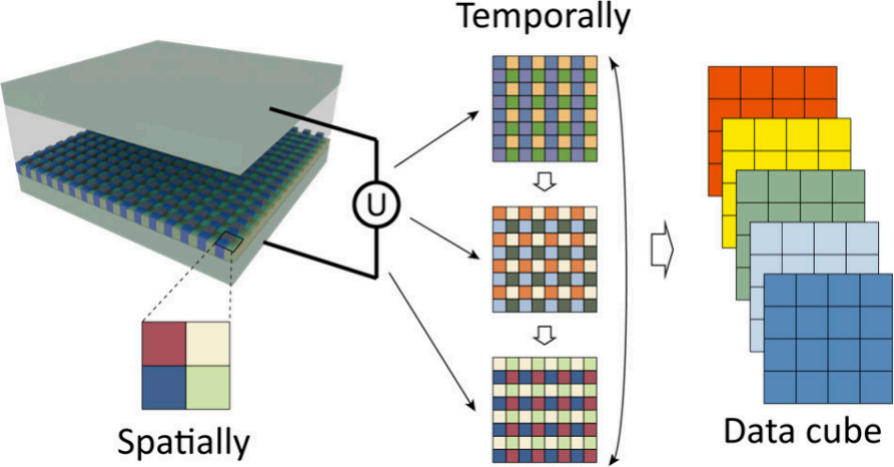

Existing miniaturized spectral imagers have mutual restrictions among spatial, temporal, and spectral resolutions, making them inadequate to meet practical needs. Here, we demonstrate a spatiotemporal filter array chip to tackle the trade-off among the three resolutions for miniaturized hyperspectral imaging. A 2 × 2 Fabry–Perot filter array is embedded inside a tunable liquid crystal spectral modulator, increasing the number of filtering channels spatiotemporally in hardware, which empowers the spectral imager with high spatial, temporal, and spectral resolutions simultaneously. The tunable filter is of transmission type with the advantages of easy fabrication and large modulation. Experimental results show the proposed spectral imager has a spectral resolution of around 0.5 nm in the visible range, with a frame rate of 60 Hz and a spatial resolution of up to 25 lp/mm without postinterpolation. The proposed filter gives the best balanced performance for spectral imaging in terms of spatial, temporal, and spectral resolutions.

Spectral cameras provide both spatial and spectral information on the scene simultaneously and are essential to fields such as healthcare,^1,2^ environmental monitoring,^3^ food safety,^4,5^ and machine vision.^6^ Spatial-scanning,^7^ spectral-scanning,^8,9^ and snapshot^10,11^ spectral cameras are popular in recent research and commercialized in industry. However, these three types of spectral cameras are restricted by drawbacks such as low luminous flux, large volume, and slow imaging speed in scanning-based spectral cameras and low spatial or spectral resolution in snapshot spectral cameras.

Recently, computational spectral imaging utilizing quantum dots,^12,13^ nanowires,^14,15^ two-dimensional materials,^16,17^ Fabry–Perot cavities,^18,19^ and metasurfaces^20,21^ has been proposed. Due to broadband optical responses with low correlation, miniaturized spectral cameras with characteristics such as high signal-to-noise ratio (SNR) and high spectral resolution can be designed.^22,23^ The key parameters for filter design in these systems are the filtering channel number and randomness or, specifically, the correlation coefficients between each filter.^24,25^ There are generally two ways to introduce low-correlation-filtering channels in design. One is to utilize reconfigurable materials such as phase change materials,^26^ Micro-Electromechanical System (MEMS),^27^ and liquid crystals^28−30^ to modulate the input signal at the time dimension, leading to a high spatial but relatively low temporal resolution. The other is to design filter arrays such as different Fabry–Perot cavities^18^ to modulate the spectrum on the spatial dimension, while interpolation algorithms are necessary to compensate for the loss of spatial resolution. Until now, high spectral, spatial, and temporal resolution have not been obtained simultaneously in portable spectral camera hardware. Even though spatiotemporally modulated filter-based spectral cameras are recently proposed to balance spectral, spatial, and temporal resolution, either their large volumes and complex structures help achieve high performances^31^ or their imaging rate is far below the video rate.^19,26,32^

Here, we propose a spatiotemporal tunable filter array chip for spectral modulation and demonstrate a portable video-rate spectral imager with high spatial and spectral resolutions. The tunable filter array chip is composed of a 2 × 2 mosaic Fabry–Perot (FP) filter array embedded within a tunable liquid crystal (LC) cell, which is set between two orthogonal polarizers. Due to the birefringence of the LC material and the polarization interference effect, the LC cell can modulate the spectral response of the input light by applying various voltages.^33,34^ Cascaded with the designed FP filter arrays, abundant modulation states are introduced into the spectral modulator with lower correlation than those of sole filter arrays or LC cells. The synergistic modulation effect empowers the proposed spectral imager with high-quality spectral images, mitigating the trade-off between spatial resolution, spectral resolution, and acquisition time. Experimental results show that this system can achieve a spectral resolution of around 0.5 nm and a spectral accuracy of around 0.2 nm in the visible range, while the spatial resolution can be up to 25 lp/mm. We evaluate its application for biological and microscopic imaging as a demonstration. Spectral video recording is conducted with the device with high repeatability for a long period at a frame rate of 60 fps, validating that our spectral camera is reliable for practical usage. This simple, cost-effective hardware shows advantages over expensive hyperspectral cameras such as high optical throughput, high SNR, and high resolution on spatiotemporal dimensions, enabling many new applications such as portable point-of-care health management and quality inspection.

The schematic of the spatiotemporal filter array is shown in Figure 1(a). The mosaic FP filters, composed of two silver (Ag) mirrors and silicon oxide (SiO_2_) layers with various thicknesses, are integrated inside a tunable LC cell, which temporally modulates the spectrum. The FP cavities with different thicknesses also introduce thickness changes in each LC cell unit to further enhance the spectral modulation (Figure 1(b)) and lower the correlation coefficient between each filtering channel. Figure 1(c) shows the workflow of the spectral imager in the experiment. To simplify the alignment process, we built the spectral imager with a relay optic to apply the spatiotemporal filter array between the monochromatic camera and imaging lens, ensuring that the spectral modulator is conjugated with the image sensor. The spectrum of a scene is temporally and spatially modulated by this spectral modulator and then captured by the camera (Figure 1(c)). Due to the FP cavity filter arrays and LC cell, high spatial and spectral resolution images can be obtained with a video-rate frame rate, surpassing the pure spectral-scanning or snapshot method. Adaptive capturing time, spatial resolution, and reconstructed spectral accuracy can also be achieved by choosing different voltage and FP-cavity filter combinations for the spectral modulator, leading to a more flexible configuration for spectral imaging (for more detailed information, see Supporting Information S1).

**Figure 1:**
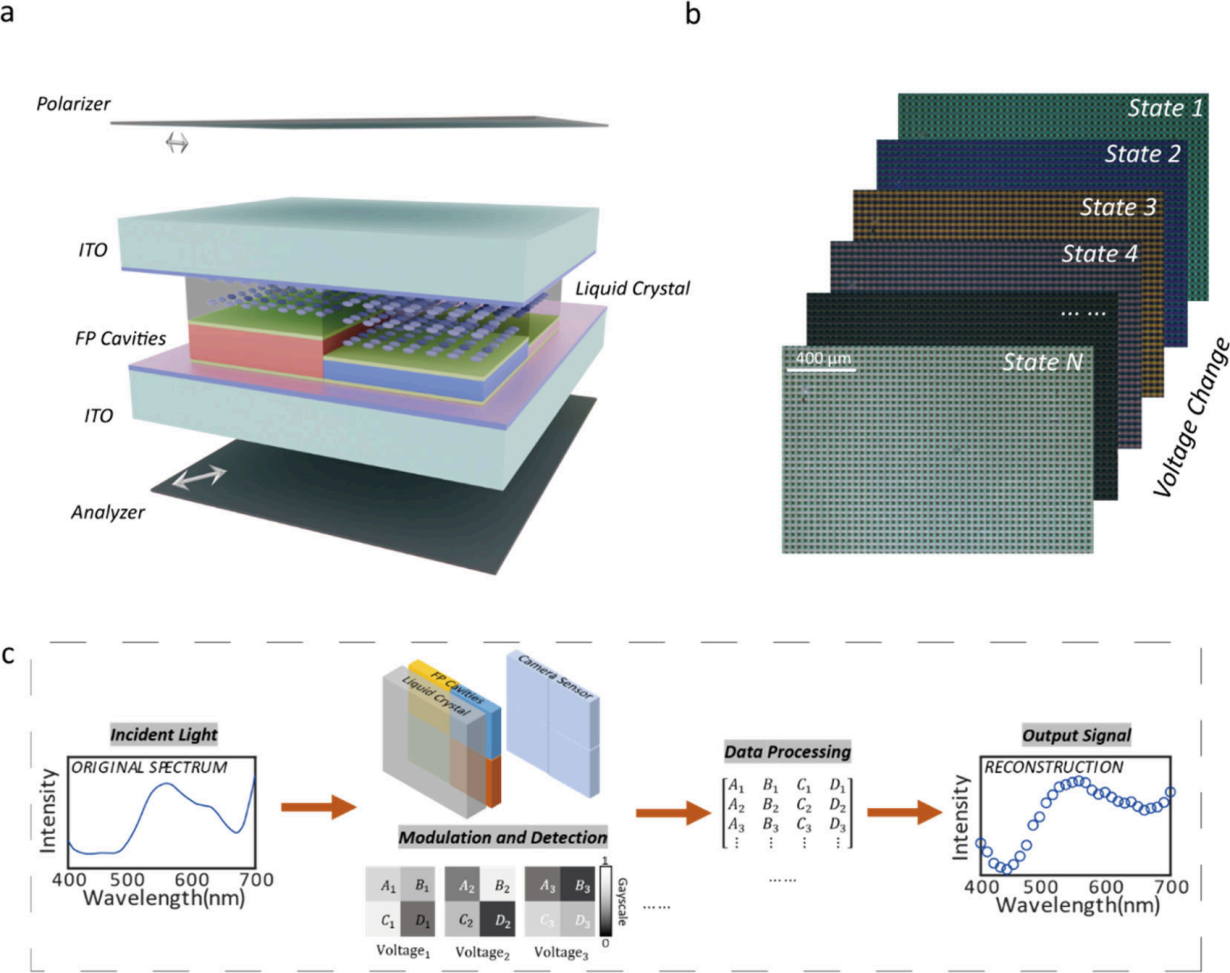
The schematic of the system. (a) Schematic of the tunable filter array. From top to bottom are the polarizer, liquid crystal cell with FP cavity array, and analyzer. The arrow indicates the polarization direction of the polarizer. (b) Color images of the spectral modulator at different states. (c) The process of data collection and processing. A, B, C, and D represent the pixel grayscale values obtained by camera sensors after different FP cavity filters, respectively, and their subscripts represent various filtering channels under different LC states.

The signal of a scene encoded by the spatiotemporal filter array and captured by the monochromatic camera can be expressed as
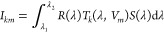
1where *I*_*km*_ represents the output signal of the camera after the *k*th filter at the *m*th driving voltage state. λ_1_ and λ_2_ are the lower and upper bounds of the working wavelength. *R*(λ) is the spectrum of the scene. *T*_*k*_(λ, *V*_*m*_) is the transmittance of the spectral modulator at the driving voltage state of *V*_*m*_ of the *k*th FP-cavity filter. *S*(λ) includes the quantum efficiency of the camera sensor and the transmittance of the imaging system, which is a constant. *T*_*k*_(λ, *V*_*m*_) and *S*(λ) compose a sensing matrix *A* which can be precalibrated. By discretizing the wavelength into *N* parts, we can express the electrical signal intensity of the camera as follows:
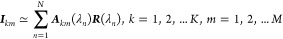
2

The input wavelength channel *N* is usually greater than the number of products of the states of the driving voltage *M* and the filter number *K*, so it is an underdetermined problem to solve ***R***. As shown in eq 3, to solve the optimization problem, in the process of minimizing the cost function, some regularization terms (***B***) are used, and the equation can be solved by a gradient descent algorithm. The driving voltage states and the FP-cavity filter array are designed based on the principle of compressing sampling, with the sensing matrix ***A*** incoherent enough for the signal measured even if it is an underdetermined system, ensuring its convergence with a nonzero error in the reconstruction (for more details, see Supporting Information S2). The Pearson correlation coefficient is used to characterize the correlation of the combinations of different voltages and filters in the spectral modulator (for more detailed information, see Supporting Information S2).
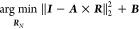
3

The transmittance of the spectral modulator *T*_*k*_(λ, *V*_*m*_) is the multiplication of the transmittance of the LC cell, *LC*(λ, *V*_*m*_), and the FP filter, *FP*(λ, *d*_*k*_). The transmittance of a nematic LC cell between two orthogonal polarizers can be approximated as sin^2^ δ/2, where δ is the phase retardation introduced by liquid crystal (for more information, see Supporting Information S3) and can be calculated as δ = 2*πn*_*V*_*m*_,λ_*d*/λ, where *n*_*V*_*m*_,λ_ changes from Δ*n* to 0 as the applied voltage increases. Here Δ*n* = *n*_*e*_ – *n*_*o*_ is the birefringence of the liquid crystal material. Hence, the transmittance of a bare LC cell is equal to sin^2^ π*n*(*V*_*m*_, λ)*d*/λ. Figures 2(a) and 2(b) show the simulated transmissive response of a bare LC cell and its correlation coefficient matrix. The correlation coefficient is close to unity between the adjacent channels, while the transmittance of the FP cavity, *FP*(λ, *d*_*k*_), varies with the thickness of the cavity *d*_*k*_ and the maximum thickness is smaller than 4 μm. The transmittance of the spectral modulator *T*_*k*_(λ, *V*_*m*_) can be expressed as *LC*(λ, *V*_*m*_) × *FP*(λ, *d*_*k*_). The thicknesses *d*_*k*_ for each filter are found through a particle swarm optimization algorithm to minimize the average correlation coefficients between different channels (for more detailed information, see Supporting Information S4). The transmittance and correlation coefficient matrix of the spatiotemporal filter after adding a 2 × 2 FP filter are shown in Figure 2(c) and Figure 2(d). The average correlation coefficient has decreased from 0.49 to 0.29, which would significantly improve the accuracy of the spectral reconstruction and enable a large compressed sampling rate.

**Figure 2:**
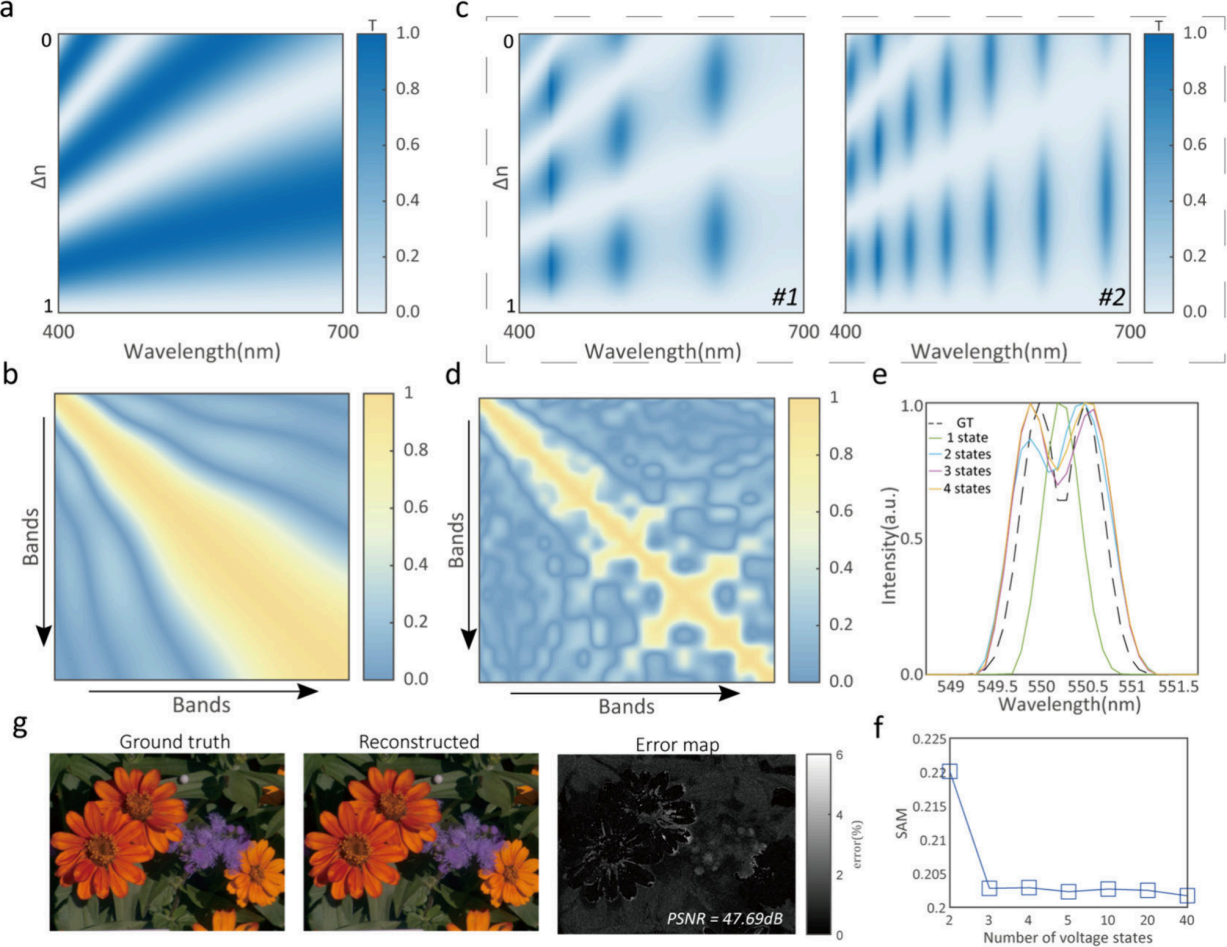
Simulated performance of the spatiotemporal tunable filter. (a) The transmittance of a bare LC spectral modulator. In the simulation, the thickness *d* of the LC cell is 4 μm, and the refractive index of LC is adopted from the reference.^35^ The *n*_*V*_*m*_, λ_ varies from 0 to Δ*n*. (b) Correlation coefficient matrix of the bare LC spectral modulator, and the average correlation coefficient is 0.49. (c) The typical transmittance of the LC spectral modulator cascaded with two FP cavities (the transmittance for the other two filters can be found in Supporting Information S4). The *n*_*V*_*m*_,λ_ varies from 0 to Δ*n*. (d) Correlation coefficient matrix of the LC spectral modulator cascaded with four FP cavities; the average correlation coefficient is 0.29. (e) Ground truth and reconstruction of double-peak Gaussian input when adopting four FP-cavity filter arrays and 1, 2, 3, and 4 voltage states for reconstruction, respectively. (f) SAM of the reconstruction of double-peak Gaussian input when adopting four FP-cavity filter arrays and different numbers of voltage states. (g) Ground truth and reconstructed images using four FP-cavity filter arrays and four voltage states. The image on the right side is the error map between the ground truth and the reconstruction. The images are adapted from ref (36). Available under CC-BY [4.0]. Copyright 2022 [David H. Foster and Adam Reeves].

We simulate the reconstruction of a double-peak Gaussian signal when taking a fixed number of FP-cavity filter arrays and different numbers of voltage states, as shown in Figures 2(e) and 2(f) (for more information, see Supporting Information S5). The relationship between the number of applied voltage states and the reconstructed spectral resolution is analyzed and shows that only three voltage states are needed to achieve a spectral resolution of 0.5 nm in a wide wavelength band. The spectral angle mapper (SAM) for the reconstruction can decrease to 0.203 with voltage states larger than three (Figure 2(f)) (the definition of SAM can be found in Supporting Information S5). The reconstruction of spectral images using hyperspectral data sets^36^ shows that a peak signal-to-noise ratio (PSNR) of 47.69 dB is achieved by using only four voltage states (Figure 2(g), more detail in Supporting Information S6). The combination of 2 × 2 filters has the least impact on spatial resolution, and spatial resolution can be further restored using the de-mosaic algorithm similar to the method of Bayer filter-based color cameras. A larger number of filters is helpful to achieve a high spectral reconstruction, but there is a greater loss of spatial resolution.

In Figure 3(a), we show the measured transmissive response for our filters under different driving voltages. (The fabrication and calibration details can be found in Supporting Information S7.) The low correlation coefficient (the average correlation coefficient is 0.34, Figure 3(b)) indicates that our spectral camera has a superior spectral reconstruction ability and high compression sampling rate. Given that the response changes more dramatically in the range from 0.8 to 4.5 V, the spectrum reconstruction is achieved by selecting the filtering states in this range.

**Figure 3:**
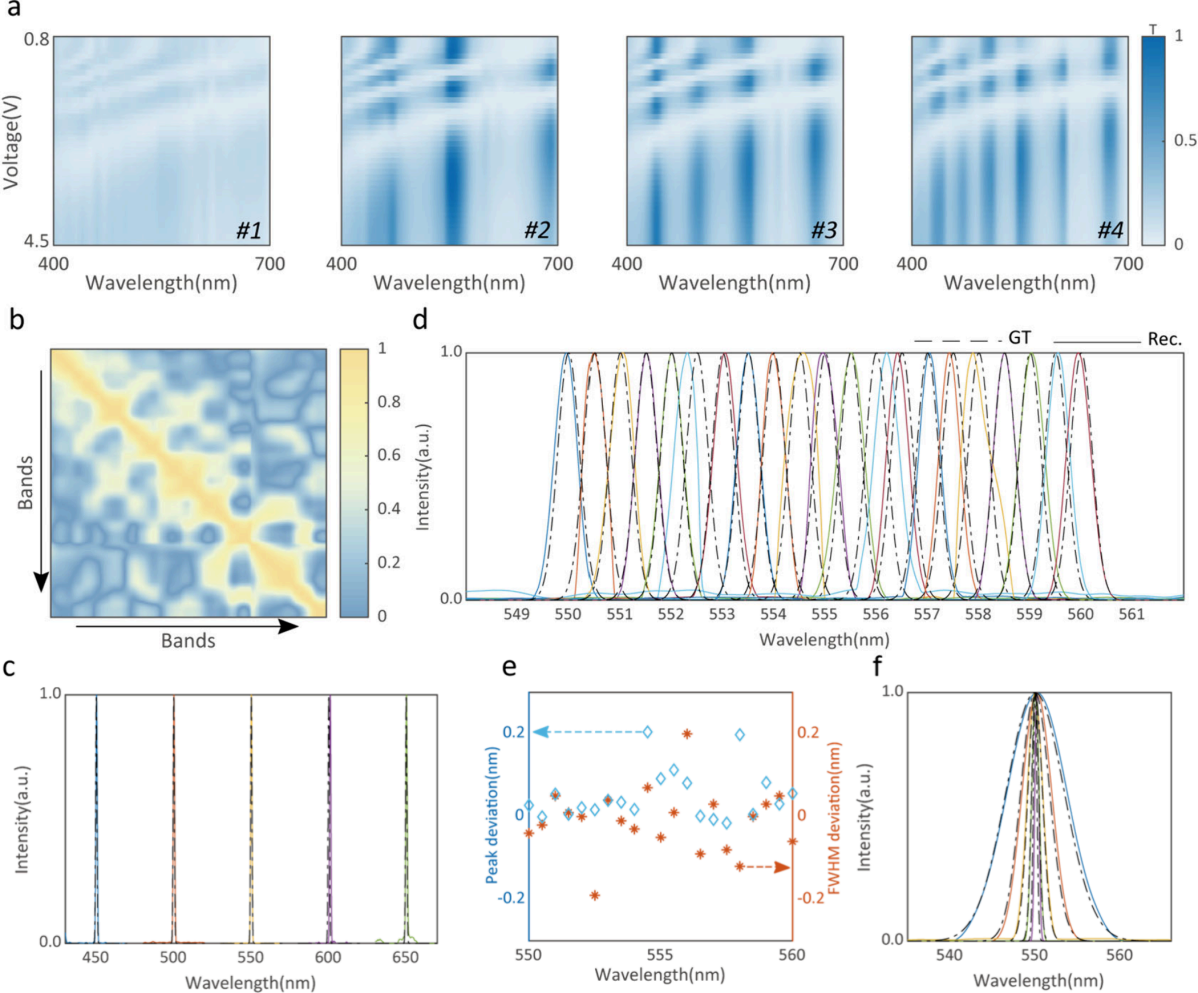
Performance of the spectral camera. (a) Transmittance of filters under different driving voltages. (b) Correlation coefficient matrix of the measured spectral modulator. (c) Reconstructed spectra of 1 nm wide peak signals at 450, 500, 550, 600, and 650 nm. (d) Reconstructed spectra of 0.5 nm wide peak signals from 550 to 560 nm. (e) Peak (left, blue diamond marks) and full width at half-maximum (fwhm) (right, red star marks) deviation of reconstructed spectra of 0.5 nm wide peak signals from 550 to 560 nm. (f) Reconstructed spectra of 0.5, 1, 2, 4, and 8 nm wide peak signals at 550 nm. The dashed line represents the ground truth of the spectra, and the solid line represents the reconstruction in (c), (d), and (f).

To show its ability for spectral imaging, we first measured several narrowband inputs with our system. The experimental details are discussed in Supporting Information S8. The reconstructed spectra are shown in Figures 3(c)–3(f), respectively. The results show good agreement with the original spectra. Figure 3(c) shows the recovered spectra in a wide range, and the ground truth of the spectra is 1 nm wide peak signals. Although the bandwidth of reconstructed spectra at some wavelengths has a deviation, the position of the peak is quite accurate. The resolution of our device is also evaluated by a series of signals with a 0.5 nm-wide bandwidth within a narrow wavelength range. The spectral camera is capable of distinguishing two peaks with a wavelength interval of 0.5 nm (Figure 3d), demonstrating we can achieve a resolution around 0.5 nm and an accuracy of 0.2 nm (Figure 3(e)). The reconstruction ability for 0.5 nm monochromatic light is also validated in Figure 3(f) with input signals with different bandwidths (for Figure 3(c) and 3(f), their deviation of the position and width between the original and reconstructed signal is also shown in Supporting Information S8).

In Figure 4, to demonstrate its practical applications, several colorful scenes are measured by our spectral camera at both microscopic (Figure 4(a) and top of Figure 4(c)) and macroscopic (bottom of Figure 4(c)) scales (for further experimental details, please refer to Supporting Information S9). The reconstructed images have a resolution of 240 × 180 pixels and show little apparent color difference compared with the original images (the color difference is mainly caused by different white balance settings of the camera). All of the reconstructed spectra fit well with the ground truth, and the average root mean square error (rmse) is less than 0.083 (Figure 4(b)). In Figure 4(c), we image a colorful biological specimen with a 4× objective (NA = 0.1) and color checkerboard with a prime lens (focal length = 25 mm). As shown in the reconstructed image, the details can be seen clearly with high color fidelity. Even with such high spectral resolution, the spatial resolution of the device can be 25 lp/mm (the microimage is enlarged by a 4× objective shown in Figure 4(d)) by imaging the resolution board with the spectral imager. This is close to the theoretical resolution of 40 μm as our filter occupies 11 × 11 pixels of the camera.

**Figure 4:**
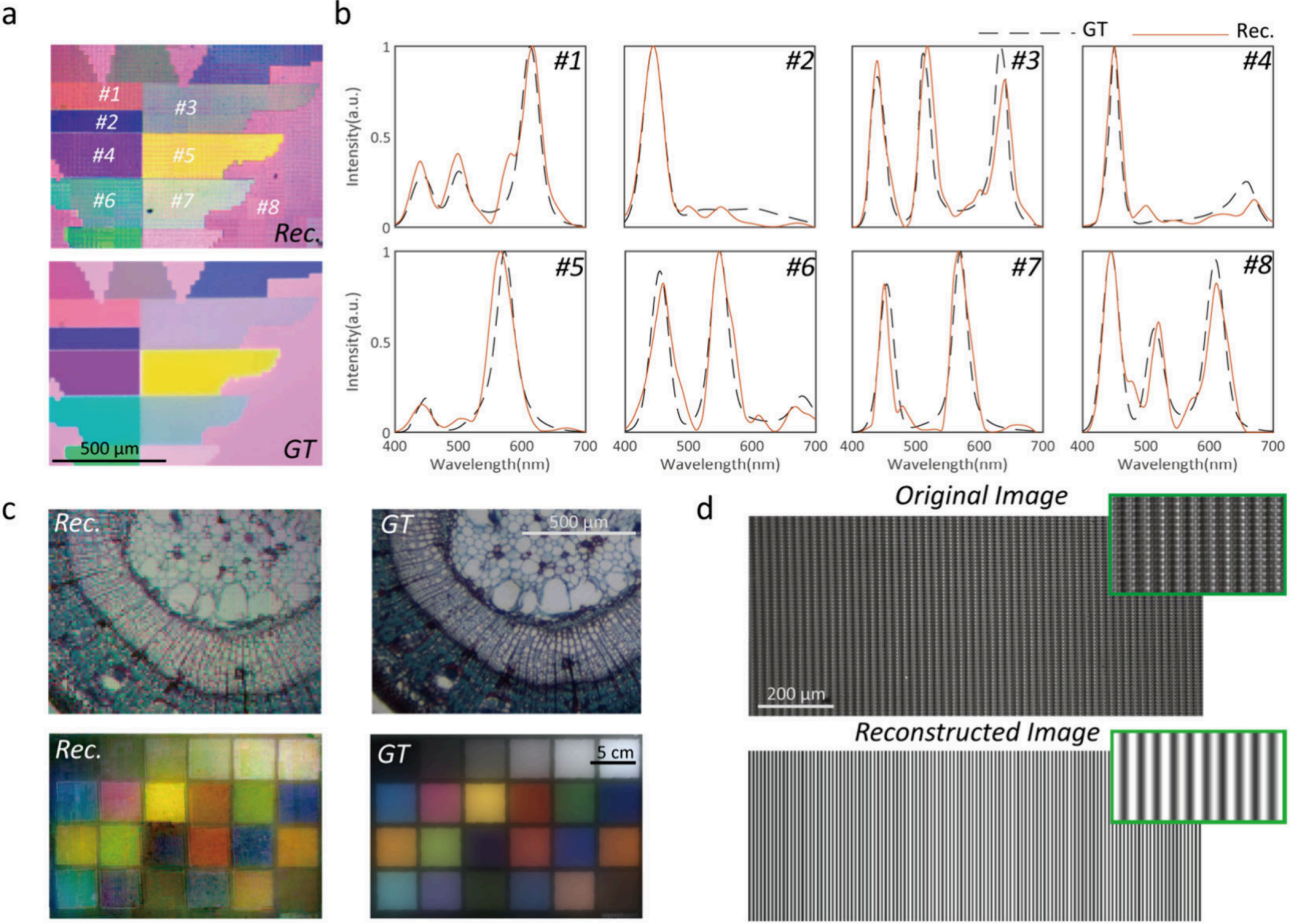
Spectral imaging for microscopic and macroscopic scenes. (a) The color image reconstructed by our spectral camera (*Rec.*) and its original color image captured by a commercial colored camera (*GT*) of a colorful pattern. (b) Reconstructed spectra (orange solid line) and its ground truth (black dashed line) of relative areas in (a). (c) The color image reconstructed by our spectral camera (*Rec.*) and its original color image captured by a commercial camera (*GT*) of different objects. (d) Original and reconstructed images of the resolution board (100 lp/mm); the inset image is an enlarged region of the reconstructed image.

Finally, we demonstrate the potential application of our spectral camera for spectral video capturing of a green paramecium. In the experiment, we switch the driving voltage between 0 and 10 V at a rate of 30 Hz, while the camera collected original spectral information on the scene at more than 200 frames per second. The driving voltage variation is shown in Figure 5(a); nodes of voltage jump are considered as the time nodes of reconstructed frames, and the reconstruction of one frame needs all data around this time node within 33.33 ms. The driving voltage of the arbitrary function generator and the equivalent spectral modulator state (represented by the corresponding voltage where the modulator shows the same intensity) within one 33.33 ms time period is shown in Figure 5(b) (details can be found in Supporting Information S10). Due to the slow transitional dynamics of the LC material, the equivalent state of the spectral modulator does not immediately change to 0 V but to the equivalent state of 2.15 V with gradual rising and falling time. Due to this feature, the camera can collect 7 to 8 frames (which depends on the frame rate of the camera) at different equivalent states for spectral reconstruction during each switching cycle. After subsequent data processing, we can reconstruct the spectral images of the scene with these intermediate frames. Figure 5(c) shows frames with a 1 s time interval, and the spectral images of all pixels at all times are reconstructed. The reconstructed spectra of the green paramecium and the background are shown in Figure 5(d), in which the green band is significantly enhanced due to the rupture of the green paramecium. The acquisition speed of spectral images can reach 60 fps if one voltage change is counted as one frame at the voltage switching rate of 30 Hz. Thanks to the capacitor nature of the LC cell, the tuning power is as low as 7.47 μW/mm^2^, making it attractive for use in portable devices.

**Figure 5:**
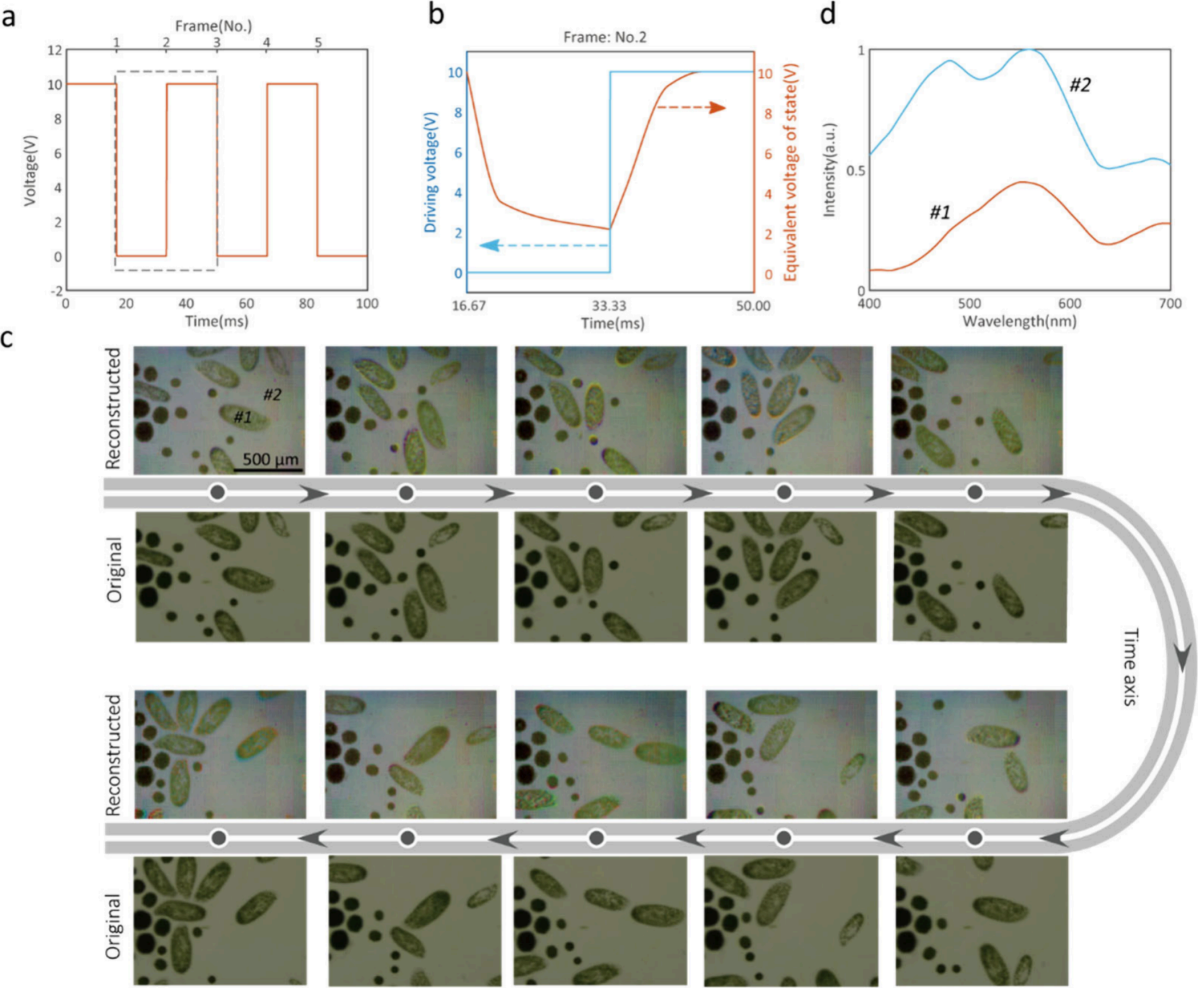
Spectral imaging for dynamic scenes. (a) Driving voltages on the spectral modulator applied by an arbitrary signal generator (bottom coordinate) and the corresponding hyperspectral image frame number (top coordinate). Each hyperspectral image is reconstructed with all data (7–8 black and white images) within one period of the voltage signal (33.33 ms). (b) Driving voltages (solid blue line) and the equivalent spectral modulator states (dashed orange line) within one voltage period shown in the dashed box in (a). (c) Reconstructed and original color images of the dynamic scenes of a green paramecium. The time interval is 1 s between each frame. (d) Reconstructed spectra of a green paramecium (red line, no. 1) and background (blue line, no. 2) at video rate labeled in (c).

In conclusion, we have demonstrated a video-rate spectral imager based on a spatiotemporally modulated tunable filter array chip with 2 × 2 Fabry–Perot filters embedded inside a thin liquid crystal cell. A spectral reconstruction accuracy of 0.2 nm and resolution of 0.5 nm are demonstrated in the experiment. High-speed hyperspectral imaging is demonstrated with a frame rate of 60 Hz. As we only use four filters in a unit, the spatial resolution of the image degrades only by a factor of 4 (in the area) as compared to the original spatial resolution, the same as with a color camera. A comparison among different approaches for spectral imaging^37,38^ is given in Supporting Information S11. By using some de-mosaic algorithms, the spatial resolution can be further enhanced. In this article, the alignment of the FP cavity array and camera pixels is a challenge, which greatly affects the final performance of the spectral camera. Integrating these reconfigurable filters directly onto the camera target surface may lead to improvements. Additionally, due to the thermo-optic effect of liquid crystals, temperature stability is also a major factor affecting device performance, which is a necessary consideration in industrial applications (for further discussion, please refer to Supporting Information S12 and S13). Owing to advancements in fabrication techniques, FP cavities of varying thicknesses can be produced in a single process using methods such as binary lithography and reflow, as described in ref (39). Considering the simple, cost-effective hardware and high optical throughput, high SNR, and high resolution on spatiotemporal dimensions, this tunable filter array provides a possible solution for spectral imaging with high spectral, spatial, and temporal resolutions, which can greatly facilitate and be extensively employed in applications like healthcare, quality control, and environment monitoring.

## Data Availability

The data that support the findings of this study are available from the corresponding author upon reasonable request.

## Supporting Information Available

Optimization of the FP cavity and voltage combinations, influence of the average correlation coefficient, simulation on the transmittance of the LC cell, the particle swarm optimization algorithm, the reconstruction of the double-peak Gaussian signal, reconstruction of the image data set, fabrication and calibration on the spectral modulator, experimental setup and reconstruction of narrowband spectra, experimental setup and reconstruction of colorful scenes, measurement and reconstruction of dynamic scenes, performance comparison, angle sensitivity analysis of the system, challenges of integrating the filter on a camera sensor (PDF)
