# Supplementary material for: A Spatiotemporal Tunable Filter Array Chip for Video-Rate Hyperspectral Imaging
Source: Nano Lett. 2025 Feb 3;25(9):3455–63. doi: 10.1021/acs.nanolett.4c05603 (PMC11887441; doi:10.1021/acs.nanolett.4c05603)
Supplement: Supplementary file 1 — nl4c05603_si_001.pdf [file nl4c05603_si_001.pdf]

**A spatiotemporal tunable filter array chip for video-rate hyperspectral imaging**

Zijian Lin[1,2,3], Tingbiao Guo[1,*], Zhi Zhang[1], Yuan Zhang[1], Haochen Chu[1], Yijia Zeng[1], Xiao Chen[1], Nan Wang[1,4] , Ruili Zhang[2] and Sailing He [1,2,4,5,*]

[1] Centre for Optical and Electromagnetic Research, College of Optical Science and Engineering, Zhejiang University, Hangzhou, 310058, People's Republic of China

[2]Zhejiang Engineering Research Center for Intelligent Medical Imaging, Sensing and Non-invasive Rapid Testing, Taizhou Hospital, Zhejiang University, Taizhou, 318000, People's Republic of China

[3] Shanghai Institute for Advanced Study, Zhejiang University, Shanghai 201203, People's Republic of China

[4] National Engineering Research Center for Optical Instruments, Zhejiang University, Hangzhou, 310058, People's Republic of China

[5] Department of Electromagnetic Engineering, School of Electrical Engineering, KTH Royal Institute of Technology, Stockholm, SE-100 44, Sweden

[*]Corresponding author: tbguo@zju.edu.cn, sailing@kth.se

# Table of Contents

S1. Optimization of the FP cavity and voltage combinations ....................3

S2. Influence of the average correlation coefficient.................................5

S3. Simulation on the transmittance of the LC cell..................................6

S4. The particle swarm optimization algorithm.......................................7

S5. The reconstruction of the double-peak Gaussian signal......................8

S6. Reconstruction of the image dataset.................................................9

S7. Fabrication and calibration on the spectral modulator .....................10

S8. Experimental setup and reconstruction of narrowband spectra.......12

S9. Experimental setup and reconstruction of colorful scenes ...............13

S10. Measurement and reconstruction of dynamic scenes ....................14

S11. Performance comparison ............................................................15

S12. Angle sensitivity analysis of the system ..........................................16

S13. Challenges of integrating the filter on a camera sensor ..................19

## S1. Optimization of the FP cavity and voltage combinations

In all simulations in this section, we set the total thickness of the FP cavity and LC cell at a constant of 4 μm. The thickness of the two silver mirrors of the FP cavity is 15 nm, and silicon oxide layers have various thicknesses (from 100 nm to 2000 nm). The thickness of the nematic LC cell varies from 2000 nm to 3900 nm, and the LC material is E7[1]. Firstly, we calculated the transmittance matrix of the 4 μm liquid crystal cell in all voltage states and its correlation coefficient matrix, and the average correlation coefficient is 0.4971 (the average correlation coefficient is defined as the average absolute value of the correlation coefficient matrix). Then we introduced different numbers of FP cavity arrays into the liquid crystal cell and the variation of the average correlation coefficient is shown in Figure S1(a). Thicknesses of FP cavities were found through a particle swarm optimization (PSO) algorithm. In the optimization, we set the average correlation coefficient as the figure of merit (FOM). The correlation coefficient will not decrease indefinitely with an increase in the number of FP cavities, indicating that the optimization of the type of spectral modulator has a limitation. Smaller correlation coefficients may be obtained by designing more complex micro-nano structures in the LC cell. Figure S1(a) indicates that a 2 × 2 mosaic FP filter array in the LC cell will achieve a sufficiently small correlation (the average correlation coefficient is 0.2887) of the spectral modulator.

We also simulated the situation where the number of FP cavities was four but with different numbers of voltage states. In the simulation, the thicknesses of the FP cavities were fixed at 2000, 1700, 960 and 660 nm. The various optimal voltage states were found by that algorithm. The average correlation coefficient was calculated and shown in Figure S1(b). As the number of voltage states increases, the average correlation coefficient will decrease to a certain extent, indicating that it is unnecessary to use all voltage states to obtain a smaller correlation coefficient. A smaller correlation coefficient represents a larger compression sampling ratio, indicating that fewer measurements can meet the accuracy requirements for reconstruction. Adaptive number of FP cavity and voltage combinations will be helpful in different application situations.

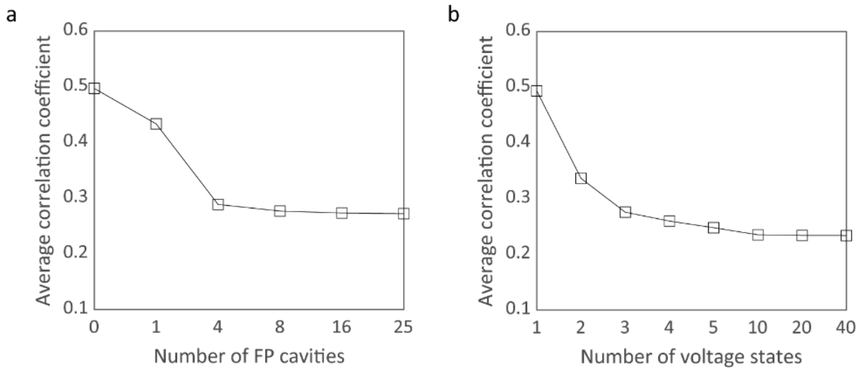


**Figure S1. The variation of the average correlation coefficient with different configurations.** (a) The average correlation coefficient varies with different numbers of FP cavities in all voltage states (The total number of voltage states is 101). (b) The average correlation coefficient varies with different numbers of voltage states in the configuration of four FP cavities.

## S2. Influence of the average correlation coefficient

The definition of the Pearson correlation coefficient is shown in Formula S1. Correlation coefficients between the wavelength bands $i$ and $j$ can be expressed as:

$$r_{ij} = \left| \frac{\sum_{m=1}^{M} \sum_{k=1}^{K} (i_{km} - \bar{i})(j_{km} - \bar{j})}{\sqrt{\left(\sum_{m=1}^{M} \sum_{k=1}^{K} (i_{km} - \bar{i})^2\right)\left(\sum_{m=1}^{M} \sum_{k=1}^{K} (j_{km} - \bar{j})^2\right)}} \right| \quad (S1)$$

where $i(j)_{km}$ is the transmittance at the applied voltage state of $m$ and FP-cavity filter of $k$. $\bar{i}(\bar{j})$ is the average transmittance. The closer the value of $r$ to zero, the lower the correlation between filtering channels is. Figure S2 is the simulation of the influence of the average correlation coefficient on reconstruction accuracy. We simulated the datasets[3] using measurement matrices with the same state number (that is, four FP filters and each with four different voltage states). The different correlation coefficients are obtained by changing the combinations of the voltage states. It can be concluded from Figure S2 that as the correlation coefficient of the measurement matrix increases, the reconstruction error also increases, validating the correction of choosing the average correlation coefficient as the FOM.

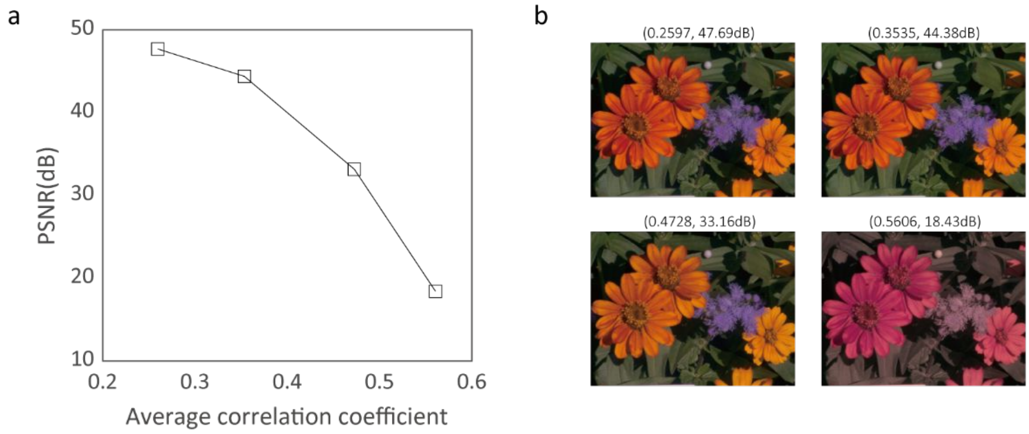


**Figure S2. The influence of the average correlation coefficient on reconstruction accuracy.** (a) Peak signal noise ratio (PSNR) as a function of the average correlation coefficients of measurement matrices. (b) The data and their reconstruction results for (a). The numbers in the brackets refer to the average correlation coefficient and PSNR. The images are adapted from [3]. Available under a CC-BY [4.0]. Copyright [2022] [David H. Foster and Adam Reeves].

## S3. Simulation on the transmittance of the LC cell

All simulations were completed on MATLAB R2023a. The transmittance of a nematic LC cell and two orthogonal polarizers can be derived through the Jones matrix:

$$T_{LC} = G_3 \cdot G_2 \cdot G_1 \cdot I_{in} \text{(S2)}$$

In the formula S2, $G_1$ and $G_3$ are the Jones matrix of the polarizer and analyzer. $G_2$ is the Jones matrix of the LC cell. The polarizer, analyzer, and LC cell are placed at 0, 90 and 45 degrees respectively. $I_{in}$ is the Jones vector of the incident light and is assumed to be $\left[1/\sqrt{2}\,;1/\sqrt{2}\right]$. The Jones vector of exit light $T_{LC}$ is:

$$T_{LC} = \frac{\sqrt{2}}{4} \cdot \begin{bmatrix} 0 & 0 \\ 0 & 1 \end{bmatrix} \begin{bmatrix} 1 + \mathrm{e}^{\mathrm{i}\delta} & 1 - \mathrm{e}^{\mathrm{i}\delta} \\ 1 - \mathrm{e}^{\mathrm{i}\delta} & 1 + \mathrm{e}^{\mathrm{i}\delta} \end{bmatrix} \cdot \begin{bmatrix} 1 & 0 \\ 0 & 0 \end{bmatrix} \cdot \begin{bmatrix} 1 \\ 1 \end{bmatrix} = \frac{\sin \delta/2}{\sqrt{2}} \begin{bmatrix} 0 \\ \sin \delta/2 - \mathrm{i}\cos \delta/2 \end{bmatrix} \text{(S3)}$$

The polarized transmittance of the spectral modulator can be expressed as:

$$|T_{LC}|^2 = \sin^2 \frac{\delta}{2} \text{(S4)}$$

where $\delta$ is the phase retardation introduced by liquid crystal and can be calculated as $\delta = 2\pi n_{V_m,\lambda} d/\lambda$, where $n_{V_m,\lambda}$ changes from $\Delta n$ to 0 as the applied voltage increases. While the transmittance of the FP cavity $FP(\lambda, d_k)$ varies with the thickness of the cavity $d_k$, the transmittance of the spectral modulator $T_k(\lambda, V_m)$ can be expressed as:

$$T_k(\lambda, V_m) = LC(\lambda, V_m) \times FP(\lambda, d_k) = \sin^2 \frac{\pi n(V_m, \lambda) d}{\lambda} \times FP(\lambda, d_k) \text{ (S5)}$$

In the simulations of Figure 2a and Figure 2c, we divided $n$ into 100 parts from $\Delta n$ to 0 evenly.

## S4. The particle swarm optimization algorithm

The particle swarm optimization (PSO) algorithm was achieved through a homemade MATLAB script to find the optimal parameters. A solution space was obtained by scanning the thickness parameters of the FP cavity in FDTD and the LC layer in MATLAB. Then we run PSO:

1) Initialize particle swarm parameters including randomly setting the velocity and position of particles and their boundaries.

2) Calculate the fitness of the particle. Here, the fitness is defined as the average correlation coefficient of the particles.

3) Update the velocity and position of particles under the restriction of the parameters.

4) Update individual optimal solutions (filter states) and group optimal solutions (the average correlation coefficient).

5) Judge the stop condition and return the optimal solution.

In Section 1, thicknesses of FP cavities and states of voltage are regarded as particles respectively. In Figure 2 in the main text, two etching depths are regarded as the particles to simulate the etched thicknesses during the double etching process for fabrication (see etching processes in section 7). The solution of the PSO algorithm is (1040, 300), so that the four thicknesses of the FP cavities are 2000, 1700, 960 and 660 nm (since the original thickness of the $SiO_2$ in the FP cavity is 2000 nm). Figure S3 shows the transmittance of the four filters. In Section 5, only voltage states are regarded as particles.

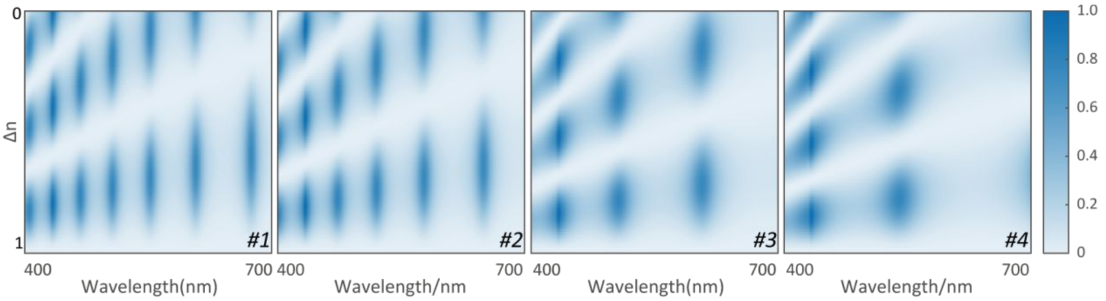


**Figure S3. The transmittance of the LC spectral modulator cascaded with four FP cavities.** Thicknesses (FP cavities) of #1, #2, #3 and #4 are 2000, 1700, 960 and 660 nm.

## S5. The reconstruction of the double-peak Gaussian signal

We simulated the reconstruction of the double-peak Gaussian signal in the wavelength range from 500 to 600 nm. The spectrum with two peaks was generated by the "normpdf" command in MATLAB. In this simulation, voltage states were selected through the PSO algorithm and the CVX toolbox[2][1] was used to reconstruct the spectra. We used equation (3) in the main text to solve for one thousand points within the wavelength range (0.1 nm interval within 100 nm band) and set the regularization coefficient to 0.

Spectral Angle Mapper (SAM) is used to reflect the reconstruction performance. It is widely used in remote sensing to measure the similarity of two spectra. The spectral similarity can be obtained by considering each spectrum as a vector in $q$ dimensional space, where $q$ is the number of bands. The SAM determines the spectral similarity between two spectra (***X*** and ***Y***) by calculating the angle between the two spectra, treating them as vectors in a space with dimensionality equal to the number of bands. It is defined as:

$$SAM(\boldsymbol{Y}, \boldsymbol{X}) = \cos^{-1}\left(\frac{\boldsymbol{Y}^T\boldsymbol{X}}{||\boldsymbol{Y}||\,||\boldsymbol{X}||}\right) \text{(S6)}$$

## S6. Reconstruction of the image dataset

Firstly, we interpolated the wavelength of the HIS datasets[3] to 400-700 nm. Then we reconstructed the spectrum by using different numbers of voltage states using the CVX toolbox[2] via a homemade MATLAB script. After obtaining the spectra for each pixel, the color image is recovered via a homemade MATLAB script (see section 9). The PSNR was calculated via "psnr" command in MATLAB.

## S7. Fabrication and calibration on the spectral modulator

A 15-nm Ag layer using DC sputtering was first deposited on an ITO substrate. A 2000 nm $SiO_2$ thin film was then deposited by using plasma-enhanced chemical vapor deposition (PECVD). Afterward, UV photolithography and inductively coupled plasma (ICP) dry etching were conducted two times to obtain the pattern (the size of a single FP filter is 20 μm on lateral dimensions, see Figure S4 (a)). The etching depths were 860 and 950 nm, respectively. Hence, the remaining $SiO_2$ for the four filters were 190, 1050, 1140 and 2000 nm. Subsequently, a 15-nm Ag layer was deposited by the DC sputtering, and then a polyimide (PI) layer was spun coated on the FP cavity arrays. Another substrate was fabricated via spin coating PI on ITO. Two substrates were mechanically rubbed to orientate the LC in a parallel configuration, respectively. 4-μm spacers and UV glue were used to control the gap of the LC cell and glue the two substrates. E7[1] was infused into the LC cell through capillary action on a hot plate. Finally, two wires were attached to the two substrates of the spectral modulator. The simulated transmittance results of the fabricated four filters are shown in Figure S4 (b).

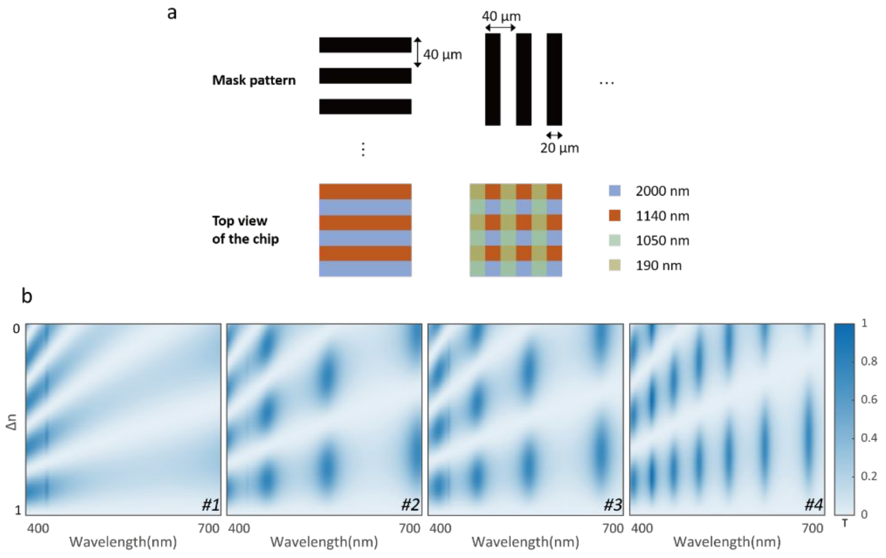


**Figure S4. (a). The schematic of the two etching processes. (b) The simulated transmittance of the fabricated spectral modulator.** Thicknesses (FP cavities) of #1, #2, #3 and #4 are 190, 1050, 1140 and 2000 nm.

In the calibration process, different monochromatic lights used for calibration were

generated by a monochromator (Microenerg (Beijing) Technology Co., Ltd, CME-TLSX300F-3G, 2-nm FWHM from 390 to 710 nm with 2-nm step size). The monochromatic light first passed a beam splitter. One part of the light was measured by a power meter (Thorlabs, PM100D and S120VC). Another part was directly illuminated onto the filter chip. In the calibration system, the spectral modulator was conjugated with the camera (HIKROBOT, MV-CS016-10UM) via a relay lens (Thorlabs, MAP107575-A). Images of FP cavity patterns were captured while changing the driving voltage of the LC cell, under different wavelength light illuminations. The spectral modulator was driven by an arbitrary function generator (LBTEC, LCVRC-2K25, a square wave with a frequency of 2 kHz from 0 to 10 V). The schematic diagram of the calibration optical path is shown in Figure S5. All instruments were controlled through a homemade LABVIEW script.

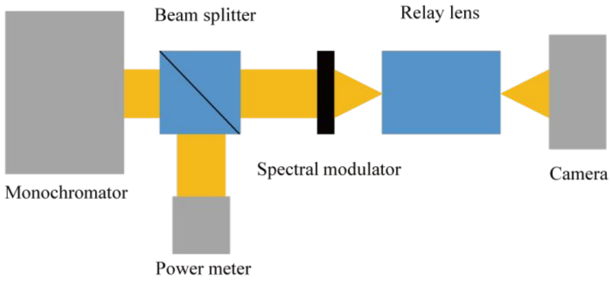


**Figure S5. Schematic diagram of the calibration optical path.**

## S8. Experimental setup and reconstruction of narrowband spectra

The experimental optical path diagram is shown in Figure S5. The narrowband spectra were generated by the monochromator. The peak width of the monochromatic light can be set between 0.5 nm and 8 nm by adjusting the width of the entrance slit. All spectra in Figures 3d, 3c and 3f in the main text were reconstructed with an accuracy of 0.1 nm within a bandwidth of 40 nm near the center wavelength, which means 401 points need to be reconstructed in the given wavelength band. A gradient descent algorithm via a homemade MATLAB script was used to solve the equation (3) in the main text and all voltage points ranging from 0.8 V to 4.5 V with intervals of 0.1 V were used. Finally, rmse was calculated via "rmse" command in MATLAB. Figure S6 shows the deviation of the position and width between the original and reconstructed signal for Figures 3(c) and 3(f) in the main text.

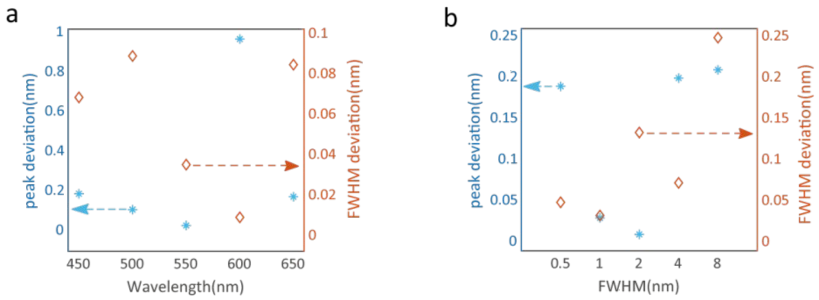


**Figure S6. Deviation of the peak and width between the original and reconstructed signal.** Peak (left, blue star marks) and FWHM (right, red diamond marks) deviation of reconstructed spectra of (a) 1-nm wide peak signals from 450 nm to 650 nm and (b) 0.5-nm, 1-nm, 2-nm, 4-nm and 8-nm wide peak signals at 550 nm

## S9. Experimental setup and reconstruction of colorful scenes

Based on the calibration optical path, we have modified the optical path at the light source end according to practical applications. The experimental optical path diagram is shown in Figure S7. For microscopic applications, we used a 4× objective lens (Olympus, 1-U2B222, NA = 0.1, working wavelength: 400 - 700 nm) and a tube lens (Thorlabs, TTL200-A) to project the image of the object onto the spectral modulator and the sensor of the color camera (ZWO, ASI662MC), respectively. And then a relay lens (Thorlabs, MAP107575-A) projected the mixed image (spectral modulator arrays and object) onto the black and white (B/W) camera (HIKROBOT, MV-CS016-10UM). For macroscopic applications, the objective lens and tube lens were replaced by a 16-mm prime lens (HIKROBOT, MVL-KF1628M-12MPE).

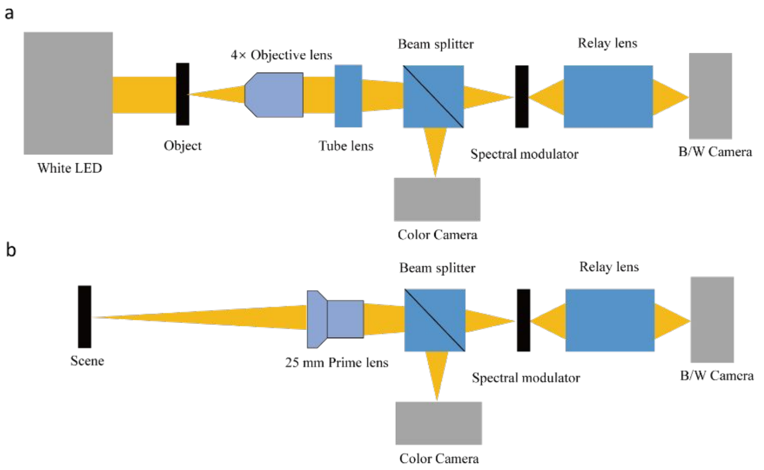


**Figure S7. Schematic diagram of the measurement optical path.** (a) Optical path of microscopic measurement. (b) Optical path of macroscopic measurement.

Reconstruction of the spectral image was conducted by solving equation (3) in the main text with a gradient descent algorithm via a homemade MATLAB script. The wavelength interval was set as 1 nm within a bandwidth of 300 nm from 400 to 700 nm (301 points). All voltage points ranging from 0.8 V to 4.5 V with intervals of 0.1 V were used. Next, we converted the spectra into tristimulus values with a homemade MATLAB script. Tristimulus values were then converted into chromaticity values in sRGB space using "xyz2rgb" command in MATLAB and the colorful images were shown in Figures 4(a) and 4(c) in the main text.

## S10. Measurement and reconstruction of dynamic scenes

Extra calibration was needed for dynamic scenes as the transmittance of the spectral modulator was different at different transition times. The calibration optical path was shown in Figure S5 and an arbitrary function generator was set to transition from 0 V to 10 V at a frequency of 30 Hz. Meanwhile, the exposure time of the B/W camera was set to less than 2 milliseconds to ensure the camera always recorded at maximum frame rate (~200 fps). Record the transmittance of the device at each voltage for a long time under each monochromatic light. The driving voltage of the arbitrary function generator and the equivalent spectral modulator state in the time dimension are shown in Figure 5(a) and 5(b) in the main text.

The process to identify the sensing matrix of each frame is as follows:

(1) **Get the stationary response of the LC filter one by one.** We first shined a monochromatic light through the LC filter chip and recorded the signal with a camera. After applying different voltages (from 0 to 10 V) on the filter chip one by one, we can get the response library between the applied voltage and the response intensity for our LC filter.

(2) **Get the dynamic response of the LC filter within a period.** We added a 30 Hz square voltage signal (0-10 V) on the LC filter and recorded the signal with the same configuration. The camera can capture 7-8 frames in a period (33ms).

(3) **Registration dynamic response with the stationary response.** We find the closest response in the response library for each frame captured dynamically and assign the corresponding voltage as the equivalent spectral modulator state for each frame.

Measurement was run with the optical path as Figure S7(a). The camera recorded the information and time of each dynamic scene at maximum speed. Finally, data at different times during the calibration and measurement process were aligned, the reconstruction of the spectral image by solving equation (3) in the main text with a gradient descent algorithm via a homemade MATLAB script (1-nm accuracy, 400 to 700 nm, 301 points). The calculation of color images was the same as the method in section 9.

## S11. Performance comparison

Compared with the filter array-type spectral imaging system, the proposed filter has a higher spatial resolution due to fewer filter numbers in a unit cell. Due to the simple fabrication process and configuration, it shows low cost and simple system complexity, and power comsuption, this is especially important for portable devices. Moreover, the response time of the proposed filter can reach to video rate with the dynamic capturing ability, compared with other tunable approaches.

Table 1. Comparison between other video-rate spectral imaging systems and ours

| Strategy | Spectal resolution | Spatial resolution | Temporal resolution | Fabrication | Cost | Ref. |
|---|---|---|---|---|---|---|
| Freeform shaped meta-atoms onto image sensor | 26 bands<br>450-700 nm | 256×256 | Snapshot | Difficult | Medium | [4] |
| FP filters array onto image sensor | 20 bands<br>450-650nm | 1920×1080<br>(for 34.4 fps)<br>3 px for 3 dB contrast (AI method) | 206.6 fps (for 640×480) | Difficult | High | [5] |
| Color camera with single LC | 30 bands<br>410-700 nm | 400-700 | 8 fps | Easy | Low | [6] |
| Broadband modulation materials onto image sensor | 96 bands<br>400-1700 nm | 1024×1024 | 124 fps | Difficult | High | [7] |
| Different thickness of LC and FP cavity array | 0.5 nm<br>(best performance)<br>400-700 nm | 240×180<br>1920×1080<br>(potential) | 60 fps | Easy | Low | This work |

## S12. Angle sensitivity analysis of the system

The spectral modulator is sensitive to the angle of the incident light. In our system, we used a relay lens (f=75 mm, diameter=25 mm) to re-image the object onto the sensor. The camera sensor is a Sony IMX273, with a sensor size of 3.6×4.8 mm, and the length of the diagonal is 6 mm. The FOV of our system is mainly restricted by the relay lens and the camera sensor size and the calculated FOV value is below 5 degrees. In this case, we measure and calculate the sensitivity of the liquid crystal layer and an FP cavity layer separately. It is found that within 5 degrees, the angle sensitivity for both LC and FP cavities can be ignored. Therefore we didn't consider the angle effect during the calibration and measurement process. The detailed measurement and simulation are shown below:

**(1) Angle sensitivity of the liquid crystal layer.** We built a setup to measure the angle sensitivity of the LC material. The measurement system is shown in Figure A. In this configuration, we use an integrating sphere (to produce uniform and monochromatic light of 630 nm) as the light source. A bare LC cell (without FP cavities but with an analyzer and polarizer as a spectral modulator) is put at the location of the front focal plane of a relay lens (the same as the one used in our paper). Then the image (modulated by the LC cell) is imaged by the relay lens onto a camera. We normalize the intensity of the monochromatic images captured by the camera and the results indicate that the liquid crystal has good spatial uniformity in our optical path configuration (Figure A (b)). The average intensity is 0.9769 and its variance is 0.0054. This uniform intensity profile indicates that the angle sensitivity of LC can be ignored within 5 degrees.

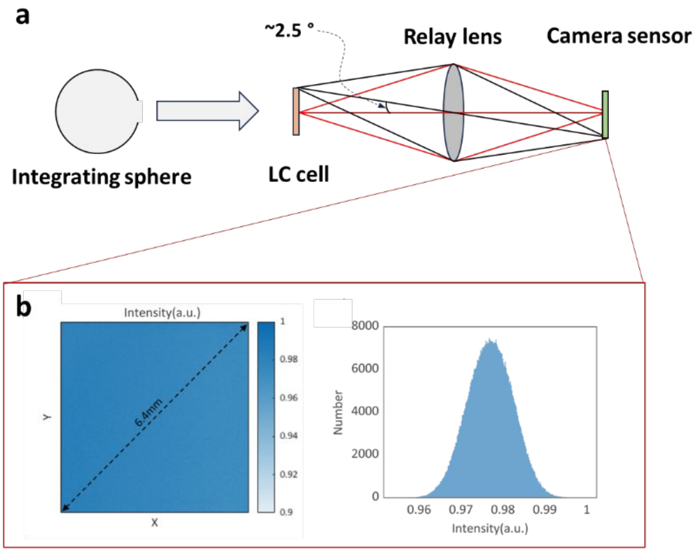


**Figure S8.** (a) Optical setup for LC angle sensitivity measurement. From left to right are integrating sphere light source, the LC spectral modulator, the relay lens, and the camera sensor. The length of the diagonal of the camera sensor is 6 mm. The aperture diaphragm is the outer shell of the relay lens with a diameter of 20.46 mm. The focal length of the relay lens is 75 mm, providing a one-to-one imaging ratio. The incident angle of the principal ray is ~2.5 degrees. (b) The intensity profile and the statistics of the intensity profile caputred by the camera sensor.

**(2) Angle sensitivity of the FP cavity.** We used the finite-difference time-domain (FDTD) method to analyze the angle sensitivity of FP cavities. The simulated results are shown below. It indicates the FP cavity is angle-insensitive within 5 degrees with negligible wavelength shift (≤0.5 nm).

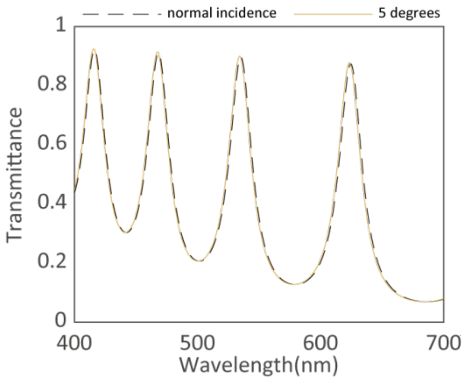


**Figure S9. Analysis of angle sensitivity of FP cavities.** Transmittance of FP cavity with

incident light at 0 and 5 degrees. The thickness of the FP cavity is 1200 nm.

In conclusion, it is found that within 5 degrees, the angle sensitivity for both LC and FP cavities can be ignored. As a result, the response spectrum of our spatiotemporal filter (FP cavities integrated into the LC cell) can be constant in this FOV region.

## S13. Challenges of integrating the filter on a camera sensor

Integrating the reconfigurable filters directly onto the camera is our next goal. For this, three challenges need to be considered:

(1) **Temperature sensitivity.** The LC material is very sensitive to the temperature. As there exists some temperature fluctuation on a camera during the capturing process, a temperature monitoring and controlling unit needs to be integrated into the camera together with our filter.

(2) **Angle sensitivity.** For practical applications, especially integrating our chip on a camera sensor, the FOV is usually larger than the one in our 'relay system'. Hence the angle sensitivity of the LC materials needs to be considered. To tackle this problem, mature approaches in LCD products such as using low birefringence LC material, and using multi-domain vertically aligned (MVA) technology.

(3) **Pixel alignment.** FP filters need to be aligned precisely with the pixels in a camera. For mass production, this can be completed during the sensor manufacturing process, similar to directly integrating Bayer filters onto the sensor.

## References


[1]. Li, J.; Wen, C. H.; Gauza, S.; Lu, R. B.; Wu, S. T. Refractive Indices of Liquid Crystals for Display Applications. *J. Disp. Technol.* **2005**, *1* (1), 51-61. DOI: 10.1109/Jdt.2005.853357

[2]. Grant M, Boyd S. CVX: Matlab software for disciplined convex programming, version 2.0 beta. https://cvxr.com/cvx, (2013).

[3]. Foster DH, Reeves A. Colour constancy failures expected in colourful environments. *Proc Biol Sci* **289**, 20212483 (2022).

[4]. Yang, J. W.; Cui, K. Y.; Huang, Y. D.; Zhang, W.; Feng, X.; Liu, F. Deep-learning based on-chip rapid spectral imaging with high spatial resolution. *Chip* **2023**, *2* (2), 100045.. DOI: ARTN 10004510.1016/j.chip.2023.100045.

[5]. Yako, M.; Yamaoka, Y.; Kiyohara, T.; Hosokawa, C.; Noda, A.; Tack, K.; Spooren, N.; Hirasawa, T.; Ishikawa, A. Video-rate hyperspectral camera based on a CMOS-compatible random array of Fabry–Pérot filters. *Nat. Photonics* **2023**, *17* (3), 218-223. DOI: 10.1038/s41566-022-01141-5.

[6]. Lin, Z. J.; Guo, T. B.; Chen, X.; Tian, J. H.; Zhang, Z.; He, S. L. Low-cost, high-speed multispectral imager via spatiotemporal modulation based on a color camera. *Opt.*

*Express* **2023**, *31* (25), 42613-42623. DOI: 10.1364/Oe.508150

[7]. Bian, L. H.; Wang, Z.; Zhang, Y. Z.; Li, L. J.; Zhang, Y. N.; Yang, C.; Fang, W.; Zhao, J. J.; Zhu, C. L.; Meng, Q. H.; et al. A broadband hyperspectral image sensor with high spatio-temporal resolution. *Nature* **2024**, *635* (8037), 73-81. DOI: 10.1038/s41586-024-08109-1.
